# Scars of oxidative stress: protein carbonylation and beta cell dysfunction in diabetes

**DOI:** 10.3389/fendo.2025.1722623

**Published:** 2025-11-26

**Authors:** Ashley Ling, Katherine R. Schultz, Jefferson D. Knight, Colin T. Shearn, Sharon Baumel-Alterzon

**Affiliations:** 1 Department of Molecular and Cellular Endocrinology, Arthur Riggs Diabetes & Metabolism Research Institute at City of Hope, Duarte, CA, United States; 2 Department of Chemistry, University of Colorado Denver, Denver, CO, United States; 3 Department of Pediatrics, University of Colorado Anschutz Medical Campus, Aurora, CO, United States

**Keywords:** protein carbonylation, beta-cells, Nrf2, lipid peroxidation, diabetes, islet

## Abstract

Type 1 and type 2 diabetes are characterized by a profound loss of functional β-cell mass, driven by mechanisms that are still not fully understood. A spectrum of β-cell stressors drives this loss, including oxidative stress (OS). Unlike most cells, β-cells express unusually low levels of key antioxidant enzymes, rendering them highly susceptible to OS. Protein carbonylation (PC), a major hallmark of OS, is an irreversible modification that can be generated by covalent addition of lipid peroxidation products known as “reactive lipid aldehydes” (RLAs) into proteins, resulting in protein inactivation, misfolding, aggregation, degradation and formation of neo-antigens. PC plays a critical role in the pathogenesis of various human diseases, including diabetes. Increased RLAs and PC are found in islets, plasma, red blood cells and adipose tissue in diabetic patients and in diabetic rodent models. Limited studies, including ours, have globally mapped carbonylated proteins in pancreatic islets and specifically in β-cells. Yet no one has explored which proteins undergo carbonylation in human islets in diabetes and whether their carbonylation contributes to the loss of functional β-cell mass in T1D and T2D. Cells have three cellular lines of defense against accumulation of PC: antioxidant enzymes, phase I and II metabolic enzymes that detoxify RLAs, and degradation of carbonylated proteins by 20S proteasome and lysosome. Since genes encoding all three lines of defense are controlled by the antioxidant master regulator, NRF2, activating this factor might be more advantageous than using pharmacological carbonyl scavengers. Future studies should test whether NRF2 activation can effectively reduce PC and preserve functional β-cells in diabetes.

## 1 Introduction

In the United States, the prevalence of type 2 diabetes (T2D) has more than doubled in recent decades, affecting 12% of the population in 2025, while nearly two million individuals live with type 1 diabetes (T1D) (1, 2). Despite T1D arising from immune-mediated processes and T2D from metabolic dysfunction, both share a hallmark feature: a profound loss of functional insulin-producing β-cells, driven by mechanisms that are still not fully understood (3–5). A spectrum of β-cell stressors drives this loss, with glucotoxicity, glucolipotoxicity, endoplasmic reticulum (ER) stress, impaired autophagy, islet amyloid deposition, and mitochondrial dysfunction predominating in T2D, while viral infection, ER stress, and a pro-inflammatory immune environment predominate in T1D (6–10). Yet, accumulating evidence implicates oxidative stress (OS) as an additional major contributor to the pathology of both T1D and T2D (4, 11–18). OS arises when the production of reactive oxygen species (ROS) within cells exceeds the cellular capacity for detoxification (4, 19–22). ROS are produced as byproducts of cellular metabolism at nearly all cellular sites, including the cytoplasm, ER, peroxisomes, lysosomes, plasma membrane, and nucleus, with mitochondria representing the most prominent source (23). Additionally, during inflammation, ROS are generated directly by activated immune cells such as macrophages and indirectly in response to cytokine release (24, 25). Most cells possess a robust antioxidant defense system composed of enzymatic and non-enzymatic ROS scavengers, including superoxide dismutase (*SOD*), catalase (*CAT*), and the thioredoxin- and the glutathione-based systems, all regulated by the master OS regulator Nuclear Factor Erythroid 2-Related Factor 2 (*NRF2*) (4, 23, 26). Yet unlike most cells, β-cells express unusually low levels of key antioxidant enzymes, rendering them highly susceptible to OS (4, 27–29). Consequently, elevated ROS levels exert deleterious effects on β-cells, compromising their identity, function, proliferation, and survival, and ultimately diminishing functional β-cell mass (4, 15, 27–29).

High ROS levels induce irreversible damage to macromolecules by causing DNA strand breaks, lipid peroxidation (discussed further), and disruption of protein structure, stability and function (30). In addition, ROS create OS-derived post-translational modifications (Ox-PTMs) on regulatory enzymes and proteins, altering their structure, stability, activity or generating novel epitopes that are recognized as autoantigens by B and T cells (31, 32). Ox-PTMs include methionine oxidation and various cysteine modifications, such as disulfide bond formation, S-nitrosylation, glutathionylation, and persulfidation, Yet another modification, protein carbonylation (PC), is widely regarded as the central hallmark of OS (31, 33). PC is an irreversible modification that involves addition of an aldehyde or ketone into a protein, often resulting in protein inactivation, misfolding, aggregation, degradation and formation of neo-antigens (21, 31–34). For this reason, PC plays a critical role in the pathogenesis of various human diseases, including diabetes (21, 35–40). This review summarizes current knowledge on PC, addressing its biochemistry, methods of detection, impact on protein structure and function, involvement in diabetes, and potential mechanisms for its detoxification. Several reviews have previously discussed PC in the context of diabetes and metabolic diseases (36, 38, 39). However, none have specifically focused on its impact on pancreatic islets and β-cells, a gap in knowledge that this review seeks to fill.

## 2 Biochemical mechanisms and detection methods of protein carbonylation

**Figure 1:**
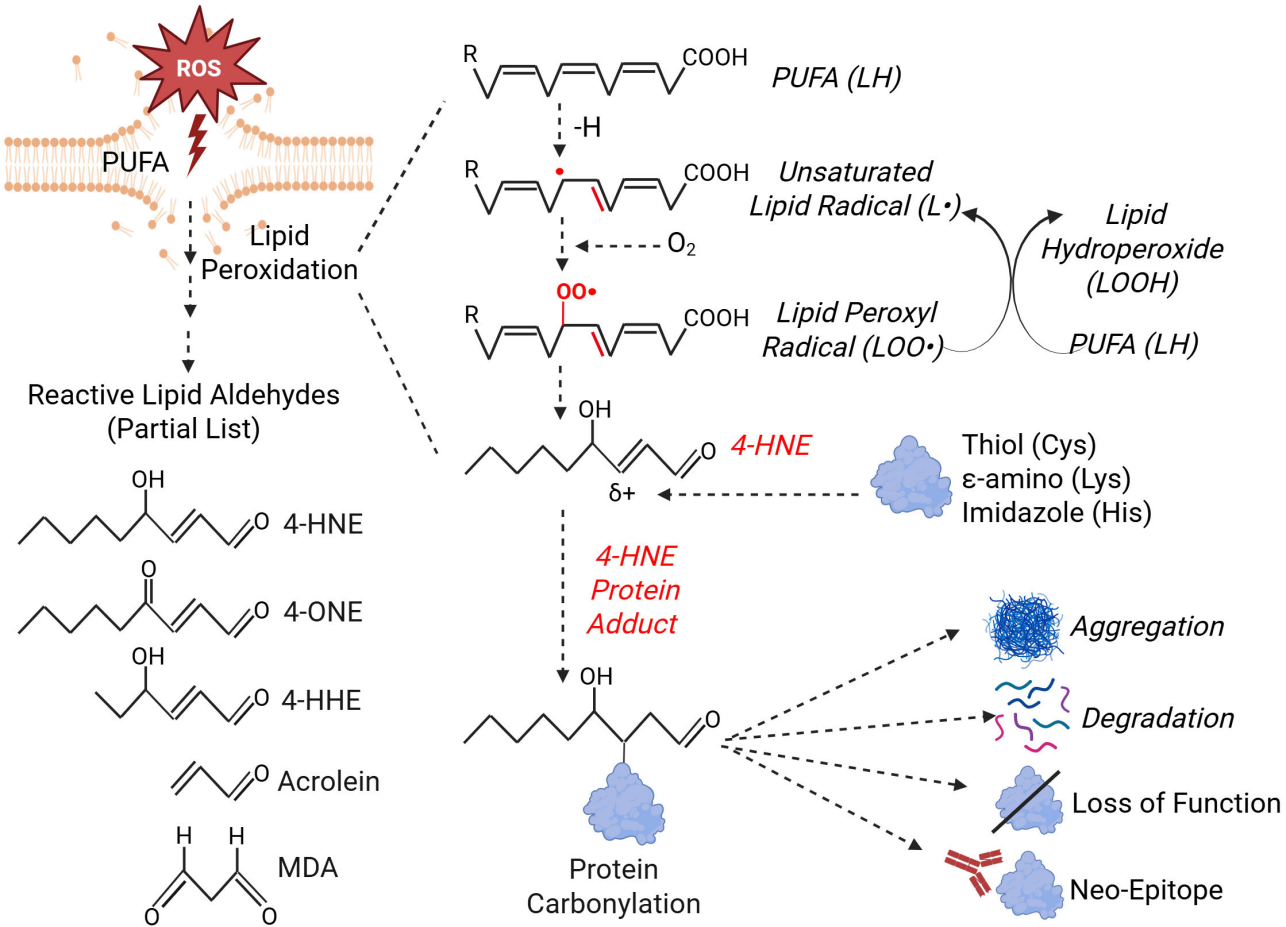
Lipid peroxidation-mediated protein carbonylation. ROS attack membrane polyunsaturated fatty acids (PUFAs), leading to lipid peroxidation and the release of reactive lipid aldehydes (RLAs). These RLAs can form protein-adducts on Cys, Lys and His residues, a process known as protein carbonylation (PC). PC can promote protein aggregation, degradation, loss of function and the formation of neo-epitopes. 4-HNE is used as an example, although similar reactions occur with other RLAs. This figure was generated using BioRender.

Upon accumulation, ROS readily attack polyunsaturated ω-3 and ω-6 fatty acids (PUFAs) in membrane phospholipids through a well-established three-step process known as lipid peroxidation. In the first step, ROS abstract a hydrogen atom from PUFA, leading to formation of an unsaturated lipid radical which can then react with molecular oxygen to form a lipid peroxyl radical. The lipid peroxyl radical can then extract a hydrogen atom from an adjacent PUFA, generating both a hydroperoxide and a new radical, thereby perpetuating the chain reaction. This process ends upon decomposition of hydroperoxides into reactive lipid aldehydes (RLAs) (41). Among the most characterized RLAs are α, β-unsaturated aldehydes such as 4-hydroxynonenal (4-HNE), 4-hydroxyhexenal (4-HHE), 4-oxononenal (4-ONE) and acrolein, as well as the dialdehyde malondialdehyde (MDA) (41, 42) (**Figure 1**). RLAs exert detrimental effects on cells by attacking lipids and nucleic acids (43, 44), yet they are mostly studied for their capacity to modify proteins (33, 39, 40, 45, 46). Mechanistically, α, β unsaturated aldehydes are electrophiles whose β-carbon bears partial positive charge and undergoes Michael addition. Nucleophiles such as the cysteine (Cys) thiol, histidine (His) imidazole, and the ϵ amino group of lysine (Lys) attack this β-carbon, forming adducts that retain the aldehyde carbonyl and thereby produce PC (**Figure 1**). In addition, α, β-unsaturated aldehydes and MDA can undergo nucleophilic attack at their carbonyl group to form a Schiff base with the ϵ-amino group of Lys (39, 45, 47, 48). While Cys, His, and Lys account for most PC events, it has been reported that, on rare occasions, arginine (Arg) can also react with RLAs through a mechanism similar to that of Lys (38, 48, 49).

Of note, although lipid peroxidation is the canonical source of PC, alternative pathways have also been observed. For example, adduction of α-dicarbonyls such as glyoxal and methylglyoxal to Lys and Arg residues is a form of PC (50). While highly relevant to diabetes, biochemically these are glycation or glycoxidation reactions, part of the Maillard reaction pathway that produces advanced glycation end products (AGEs) (51). In addition, Metal-catalyzed oxidation of Lys, Arg, Pro and Thr, cleavage of peptide bond via the α-amidation pathway, and oxidation of glutamyl residues are also potential mechanisms for PC (50, 52–54). However, limited studies have shown their physiological prevalence in β-cells in diabetes, an aspect that needs to be further explored. We will therefore focus in this review on lipid peroxidation (RLA-mediated) PC.

Detection of PC can be performed by several methods that differ in sensitivity and specificity, many of which rely on derivatization of carbonyl groups with hydrazine-based compounds to generate stable hydrazones detectable by various approaches (55). The conventional detection method relies on incubating cells with 2, 4-dinitrophenylhydrazine (DNPH) to form dinitrophenyl (DNP) adducts, which can then be detected using anti-DNP antibodies in spectrophotometric assays, ELISA, dot blot, western blot or HPLC (50, 55–58). PC can also be detected by hydrazide-based fluorescent probes, such as fluorescein-5-thiosemicarbazide (57, 58). Yet, all these methods measure the overall extent of PC without revealing which proteins are affected, the main RLAs contributing to PC formation, or the specific amino acid residues involved (57). To overcome these limitations, highly precise and sensitive mass spectrometry (MS)-based methods have been developed to enrich for cellular carbonylated proteins using biotinylated hydrazide- or hydroxylamine-based probes, followed by MS-based quantitation, a strategy known as “carbonylomics” (57, 59–68).

## 3 The impact of lipid peroxidation-mediated protein carbonylation on protein turnover and function

As amphiphilic molecules, RLAs can easily cross membranes, reaching almost any organelle within the cell, far from the site where they are formed (69). Indeed, carbonylated proteins have been found in many organelles including the cytoplasm, ER, lysosome, nucleus and the mitochondria (68, 70–76). Importantly, carbonylation is not a random process, as only up to 10% of total cell proteome is susceptible to carbonylation (52, 56, 77, 78). A protein’s susceptibility to carbonylation can be cell-type specific and depends on protein turnover rate, proximity to RLAs, and structural features of the protein that enable exposure of Cys, Lys, and His residues to this modification (52, 69, 78, 79). Due to the nature of RLAs, their attachment to a protein often leads to protein misfolding, which increases hydrophobicity, resulting in protein dysfunction, aggregation or targeting for degradation by the 20S proteasome (33, 36, 39, 80). PC can also generate new epitopes in target proteins leading to autoantigen formation (**Figure 1**) (21, 32, 46, 81).

The effect of PC on a given protein can lead to diverse biological consequences, depending on the protein’s role in the cell. In human leukemia T-cells, carbonylation of Fas cell surface death receptor activates apoptosis signal-regulating kinase 1 (ASK1), which in turn activates c-Jun N-terminal kinase (JNK) and caspase-3, leading to apoptosis (82). Carbonylation of the p46 and p54 isoforms of JNK kinase in human hepatic stellate cells activates activator protein 1 (AP-1), which stimulates the expression of procollagen type I, a fundamental step in liver fibrosis (83). In human hepatoma and breast cancer cells, carbonylation inactivates the phosphatase and tensin homolog (PTEN) tumor suppressor which facilitates the activation of protein kinase B (PKB/Akt) that stimulates cell proliferation (84, 85). Once carbonylated, the chaperone activity of heat shock protein 90 (HSP90) is impaired (86). Carbonylation of the NRF2 inhibitor, the Kelch-like ECH-associated protein 1 (KEAP1), promotes its proteasomal degradation, thereby activating the *NRF2* signaling (36, 87). The effect of PC on a given protein also depends on the site of the modified residues within the protein. For example, carbonylation of Cys^572^ in HSP90 as well as of Cys^273^ and Cys^288^ in KEAP1 have detrimental effects on these proteins (36, 86, 87). On the other hand, carbonylation at non-functional sites may not impact protein function or stability, underscoring the importance of mapping carbonylation sites and evaluating their functional consequences on protein fate.

## 4 Counteracting protein carbonylation: cellular mechanisms of defense and repair

Carbonylation is an irreversible protein modification, as no enzymatic or non-enzymatic mechanisms exist to reverse it (33, 35). Consequently, cells have evolved alternative strategies to limit the accumulation of PC. In agreement with that, exposure of intestinal enterocytes, thymocytes, hepatocytes, synovial fibroblasts, liver mitochondria, and tumor cells to 4-HNE shows that only up to 8% of total free 4-HNE modifies proteins, suggesting that the remainder is neutralized in some way (88).

The earliest cellular line of defense against PC is overexpression of antioxidant genes to prevent excessive accumulation of ROS. In line with that, Superoxide dismutase 1 (*Sod1)* knockout mice and mice expressing mutated *Sod1* exhibit increased PC in sciatic nerve and skeletal muscle, respectively (89, 90). Some antioxidant genes are specifically dedicated to inhibiting lipid peroxidation, with glutathione peroxidase 4 (GPX4) being the most extensively studied, as it utilizes glutathione to convert harmful lipid hydroperoxides into harmless lipid alcohols within cellular membranes (91). Interestingly, reduced GPX4 levels and activity are associated with complications of T1D and T2D (92, 93) and *Gpx4* haploinsufficient mice fed a high fat-high sucrose diet exhibit increased 4-HNE adducts in liver and heart (94).

The second line of defense involves direct detoxification of free RLAs by specialized phase I and II metabolic enzymes (36, 42, 95, 96). Members of the phase I metabolic enzymes use the NAD(P)^+/^NAD(P)H cofactors to oxidize RLAs to their corresponding acids or reduce them to alcohol. This includes alcohol dehydrogenases (ADH), aldehyde dehydrogenases (ALDH), aldo-keto reductases (AKR), carbonyl reductases (CBR), and cytochrome P450 (CYP) enzymes. Members of the phase II metabolic enzymes include the Glutathione S-transferases (GST) family, which conjugates RLAs with glutathione to facilitate their excretion from the body (36, 95). Inhibition in the activity of phase I and II RLA-detoxifying enzymes can contribute to β-cell dysfunction in diabetes and to diabetic complications. Accordingly, downregulation of *Cbr1* in RINm5F and HIT-T15 β-cells reduces their survival and function under glucolipotoxicity and a *CBR1* variant may contribute to T1D (97, 98). A single nucleotide polymorphism (SNP) in *CBR3* is associated with insulin resistance in T2D (99). Aldh-1, a pharmacological activator of mitochondrial ALDH2, increases β-cell function by reducing 4-HNE-mediated mitochondrial dysfunction in MIN6 and INS-1 β-cells (100, 101). Moreover, a genome-wide association (GWAS) study found an association between a SNP in *ALDH2* and T2D in East Asian individuals and in patients with diabetes-related cardiovascular diseases (102–104). Interestingly, unlike mitochondrial ALDH2, the expression of two cytoplasmic ALDH1 isoforms (ALDH1A1 and ALDH1A3) is increased in β-cells in T2D and this upregulation is associated with β-cell de-differentiation (105–108), suggesting they have additional roles beyond detoxifying RLAs. In agreement with that, both are involved in self-renewal, expansion, and differentiation in stem cells (109). Similarly, some members of the CYP and the AKR families have protective effects in diabetes and diabetes complications, whereas others worsen the disease (110–119). A SNP in the promoter of human *GSTK1–1* is associated with insulin resistance (120). Individuals with a SNP in *GSTP1* or *GSTO1*, as well as *GSTT1* and *GSTM1* null genotypes have higher risk to developing T2D and diabetes complications (121–130). GSTA4 levels are reduced in adipose tissue of obese human and mice (62, 131) and *Gsta4* knockout 129/sv mice show increased malonyl-CoA expression, mitochondrial dysfunction and obesity, contributing to development of insulin resistance (131, 132) and the microsomal glutathione transferase 1 (MGST1) plays a key role in regulating ferroptosis-mediated β-cell dysfunction in T2D and in diabetic cardiomyopathy (133, 134).

As a last line of defense, when all attempts to prevent PC fail, cells direct carbonylated proteins for degradation to reduce the accumulation of dysfunctional and misfolded proteins (33, 36, 39). The 26S proteasome is composed of two major subunits: the ATP-dependent 19S, which serves as a “gatekeeper” providing access to ubiquitinated proteins, and the catalytic 20S subunit. Due to the urgent need for their rapid degradation, oxidized proteins bypass the 19S “gatekeeper” and are directly degraded by the 20S proteasome (80, 135–137). Therefore, to increase the availability of unbound 20S proteasomes, the 26S proteasome disassembles into 19S and 20S subunits during OS (80, 136). Evidence suggests that the autophagy-lysosome system can also contribute to PC elimination to some extent (138–140). Yet, p62, a component of this system, is itself a target of carbonylation, as observed in cholestatic liver (67), suggesting that this system is impaired under certain pathological conditions. Thus, increased PC may also reflect dysfunction of the proteasome and the autophagy-lysosome systems, two phenomena linked to aging and metabolic diseases, including diabetes (33, 141–146).

Thus, considering the impairment in all three lines of defense against PC in diabetes, it is not surprising that PC plays a critical role in the pathogenesis of this disease (35–39).

## 5 Protein carbonylation in diabetes: from peripheral tissues to pancreatic β-cells

While the etiologies of T1D and T2D are somewhat different, increased OS, in particular in β-cells, plays a key role in both diseases (4, 11–18). Thus, not surprisingly, evidence suggest that PC is playing a critical role in the pathogenesis of both types of diabetes (11, 21, 35–40, 62, 68, 73, 147–164). It is expected that some proteins undergo carbonylation in both T1D and T2D. Differences may arise from distinct protein turnover and from proximity to ROS production sites. For example, the mitochondria is a major source for ROS in both types of diabetes (4, 13), resulting in enhanced carbonylation of mitochondrial proteins in both T1D and T2D (21, 68, 151, 165). However, T1D also involves an additional ROS source originating from activated circulating macrophages during immune-mediated β-cell attack (12, 13), potentially exposing additional proteins to PC. Importantly, PC may promote autoantigen formation in T1D (21, 32, 81), while evidence for a similar effect in T2D is limited. Below we summarize current knowledge on PC in T1D and T2D.

### 5.1 Protein carbonylation in type 2 diabetes

T2D patients show elevated RLAs and PC levels in plasma, urine, liver, retina, skeletal muscle, lymphocytes, red blood cells, adipose tissue and islets (148, 149, 151–164). Yet, limited studies have explored which proteins are carbonylated in T2D and how this contributes to the development of this disease (**Table 1**).

**Table 1:** Representative list of proteins undergoing carbonylation in diabetes.

Cell type	Diabetic situation	Protein name	Role	Effect of PC on the protein	Reference
Adipose tissue	Men fed an excessive caloric diet	GLUT4	Glucose transporter	Altered activity	(152)
	Diet-induced obese mice	FABP4	Fatty acid metabolism and transport	Impaired affinity for fatty acids	(62)
		ERR-γ	Mitochondrial bioenergetics	Impaired DNA binding activity	(73)
Plasma	Obese T2D patients	CPD	Carboxypeptidase	Unknown	(151)
		ASNA1	ATPase		
		VEGFR-2	VEGF receptor		
		MAP3K4	Kinase		
		MAP3K5			
		ATP1A1	Na+/K+ -ATPase		
Islets	T2D donors	ATP5b	Mitochondrial ATP synthase subunit	Unknown	(165)
Islets	Pre-diabetic NOD mice	PDIA1/2/6	Protein folding	PDIA1: potentially forming auto-antigen in T1D	(21)
		14-3-3	Scaffold protein	Unknown	
		CPA1/B2	Carboxypeptidase		
		HSPA5	Protein folding		
		AMY2A	Glycogen metabolism		
		SOD1	Antioxidant	Unknown	(68)
		GPX1			
		ATP5F1A	Mitochondrial ATP synthase subunit	Potentially inhibiting activity leading to reduced GSIS	
		INS2	Insulin		
		RAB27A	Vesicle-trafficking		
		ARF3			
		SYTL4			
MIN6 β-cells	4-HNE treatment	INS2	Insulin		
		STX5	Vesicle-trafficking		
		VAMP2			
		MUNC18-1			
		RAB3GAP1			
		AP2A2			
		DNM1L			
	Cytokine treatment	ARF1			
		RAB18			
		NSF			
		RAB7A			
		JIP4			
		SEC61A1			
		ERGIC1			
		TMED10			
		PGAP1			
		COPA			

Three of these studies have focused on adipose tissue. In the first, Boden et al. showed that carbonylation of glucose transporter type 4 (GLUT4) in adipose tissue from men fed an excessive caloric diet for one week is likely to alter its activity, as it is associated with insulin resistance (152). In another study, Grimsrud et al. showed that carbonylation of fatty acid binding protein 4 (FABP4) at Cys^117^, a protein involved in insulin resistance, is increased in mouse adipose tissue during obesity, impairing its affinity for fatty acids, potentially leading to abnormal fatty acid trafficking and lipotoxicity (62). In a third study, Hauck et al. found that carbonylation of the nuclear protein estrogen-related receptor γ (ERR-γ), a factor that promotes mitochondrial bioenergetics, in adipose tissue from diet-induced obese (DIO) mice impairs its DNA binding activity (73).

Apart from adipose tissue, T2D patients also show increased plasma PC. Accordingly, Bollineni and colleagues identified 36 proteins uniquely carbonylated in plasma from obese patients with T2D, most likely derived from the plasma, liver, platelets and the endothelium, with diverse functions including cell adhesion, signaling, angiogenesis, cytoskeletal remodeling, RNA/DNA processing, DNA repair, redox regulation, lipid and energy metabolism (**Table 1**) (151). Among these are several proteins with key roles in preventing T2D, including carboxypeptidase D (CPD) with potential role in proinsulin processing (166, 167), arsenite-stimulated ATPase (ASNA1) which participates in insulin secretion (168, 169), vascular endothelial growth factor receptor-2 (VEGFR-2) whose soluble form is elevated in plasma of T2D and is associated with insulin resistance (170), mitogen-activated protein kinase kinase kinase 4 (MAP3K4), MAP3K5 (ASK1), and sodium/potassium-transporting ATPase subunit alpha-1 (ATP1A1), each of which harbors SNPs associated with T2D (171–173). However, whether their carbonylation contributes to T2D remains to be studied.

Importantly, elevated 4-HNE levels have been reported in β-cells from DIO rodents, in islets from db/db diabetic mice, and in islets of T2D human cadaveric donors (158, 165, 174–176), suggesting of increased islet PC in T2D. In line with that, MacDonald et al. have detected five 4-HNE-protein adducts in islets of T2D donors, one of which was the mitochondrial ATP synthase β-subunit (ATP5b) (165), a key enzyme in stimulating insulin secretion (177, 178). While informative, this study relied on immunoblotting with a 4-HNE antibody, a method limited to a single type of PC and less sensitive to weakly expressed proteins, thereby restricting the number of detectable carbonylated proteins.

Further studies are still needed to explore the identity of islet proteins susceptible to carbonylation in T2D and to define the role of this PTM in T2D development.

### 5.2 Protein carbonylation in type 1 diabetes

As in T2D, increased RLAs and PC levels are found in serum and plasma of T1D patients (11, 147–150). Yet despite this strong association, global mapping of carbonylated proteins in the context of T1D has primarily focused on mouse islets (21, 68). The pioneering work of Yang et al. identified 23 carbonylated proteins in islets from pre-diabetic NOD mice (**Table 1**) (21), including members of the protein disulfide isomerase (PDI), 14-3-3, and carboxypeptidase families, as well as several metabolic enzymes. Importantly, they detected an autoreactive T-cell response in the sera from T1D patients to carbonylated prolyl-4-hydroxylase b subunit (P4Hb; also known as PDIA1), an enzyme required for the formation of proinsulin disulfide bonds (179), highlighting a potential role of PC in generating neo-antigens in T1D. Consistent with this, they also identified PC in 78 kDa glucose-regulated protein (GRP78/HSPA5) and Pancreatic amylase 2, two known autoantigens in T1D (21, 180, 181).

To elucidate which proteins are susceptible to carbonylation in β-cells in T1D settings, we recently mapped over 1000 individual carbonylated proteins in islets from pre-diabetic NOD mice side by side with MIN6 β-cells treated with 4-HNE or cytokines (68). We identify increased carbonylation in a subset of vesicle-trafficking proteins (**Table 1**), including, Sec61 translocon subunit alpha 1 (SEC61A1), ADP ribosylation factor 1 and 3 (ARF1, 3), RAB3 GTPase activating protein catalytic subunit 1 (RAB3GAP1), N ethylmaleimide sensitive factor (NSF), RAB18, member RAS oncogene family (RAB18), Syntaxin binding protein 1 (MUNC18-1), RAB7A, member RAS oncogene family (RAB7A), Syntaxin 5 (STX5), Adaptor related protein complex 2 subunit alpha 2 (AP2A2), Endoplasmic reticulum Golgi intermediate compartment 1 (ERGIC1), Dynamin 1 like (DNM1L), Transmembrane p24 trafficking protein 10 (TMED10), Post GPI attachment to proteins 1 (PGAP1) and JNK interacting protein 4 (JIP4) (68). Moreover, consistent with a previous finding (182), we observed insulin (INS2) carbonylation upon exposure to 4-HNE (68). Therefore, and not surprisingly, in agreement with the role of these proteins in maintaining β-cell function, carbonylation of these proteins was associated with impaired insulin secretion (68), supporting a previous study showing the inhibitory effect of 4-HNE on insulin secretion (183). However, whether their carbonylation participates in the development of T1D needs to be further studied.

## 6 The use of carbonyl scavengers for diabetes therapy

Although developed for other purposes, several commonly used FDA-approved drugs contain potent nucleophilic groups, such as thiol, hydrazine, primary amine, and imidazole, which make them potential carbonyl scavengers (184, 185). For example, hydralazine, a peripheral vasodilator used for hypertension, scavenges a wide range of RLAs (186). It reduces 4-HNE adducts on amyloid-β and prevents its misfolding (187), protects against allylamine-induced cardiac fibrosis and pulmonary pathology by trapping acrolein (186), and lowers MDA levels in a Parkinson’s disease cell model (188). However, although it decreases plasma lipids in diabetic rats (189, 190), it disrupts glucose homeostasis in rats and dogs (190–192), limiting its therapeutic potential in diabetes.

Edaravone, a neuroprotective drug for amyotrophic lateral sclerosis (ALS), is also an RLA scavenger (193, 194). It reduces MDA, 4-HNE, and acrolein in Alzheimer’s mouse models, in aorta of atherosclerotic mice, and in mouse ischemic brain tissue (195–198). Additionally, Edaravone lowers blood MDA, protects against retinal and renal damage, increases insulin content, reduces insulitis, and improves islet graft survival in diabetic rodents (199–204). However, it shows little effect on glucose regulation in rodents and has been linked to rare hyperglycemia events in ALS patients (199, 201–205), suggesting that, like hydralazine, it is not optimal for diabetes therapy.

Phenelzine, an antidepressant and monoamine oxidase (MAO) inhibitor, also acts as an RLA scavenger (206). It prevents 4-HNE-induced mitochondrial dysfunction in a traumatic brain injury rat model (207), reduces 4-HNE adducts in human plasma (208) and protects rat retinal cells from acrolein toxicity (209). In non-diabetic patients with depression, phenelzine lowers fasting blood glucose (210), while in various obesogenic mouse models, it reduces hepatic MDA, improves insulin sensitivity, increases plasma insulin, maintains normoglycemia, and decreases inflammation (211, 212). Its effects on β-cell function are dose-dependent, enhancing glucose-stimulated insulin secretion (GSIS) in isolated rabbit and rat islets at micromolar, but inhibiting it at millimolar concentrations (213–215). Yet, whether it can improve the lives of diabetic patients and whether its beneficial effects stem from MAO inhibition or carbonyl scavenging properties need to be further studied.

Of note, unlike carbonyl scavengers that target only a single line of defense, NRF2 regulates genes across all three defense lines, including those encoding antioxidant enzymes, RLA-detoxifying enzymes, and components of the 20S proteasome and lysosome, such as Proteasome Subunit β-Type 5 (PSMB5), PA28αβ, and Sequestosome-1 (p62) (36, 216–223). Thus, NRF2 activation may be more advantageous by simultaneously inducing all protective pathways. Indeed, pharmacological activation of NRF2 has been shown to reduce PC levels in the liver of a diet-induced nonalcoholic steatohepatitis rat model, in mouse maxillary nerves, and to lower 4-HNE levels in mouse islets (224–226). This raises the question of why activation of NRF2 through KEAP1 carbonylation (36) fails to eliminate PC in diabetes. One possible explanation is that the rate of PC formation in diabetes exceeds NRF2’s detoxifying capacity, leading to progressive accumulation of carbonylated proteins. In addition, diabetes is associated with SNPs in several RLA-detoxifying enzymes and with β-cell proteasome dysfunction (97–99, 102–104, 120–130, 227, 228), which may further compromise cellular protection. Lastly, key antioxidants may themselves become carbonylated in diabetes, as our data show increased carbonylation of SOD1 and GPX1 in islets from NOD mice (**Table 1**) (68). Future studies should test whether NRF2 activation can effectively reduce PC and preserve functional β-cells in diabetes.

## 7 Concluding remarks

In this review we summarized current findings on PC in diabetes, including recent studies in pancreatic islets and β-cells. However, it remains unclear which proteins are carbonylated in human islets and how these modifications contribute to functional β-cell loss in T1D and T2D. Future studies are needed to determine whether FDA-approved drugs with carbonyl-scavenging properties, such as phenelzine, or pharmacological activators of NRF2, can effectively reduce protein carbonylation and preserve β-cell function in diabetes.
